# Preservation of Metabolic Flexibility in Skeletal Muscle by a Combined Use of *n*-3 PUFA and Rosiglitazone in Dietary Obese Mice

**DOI:** 10.1371/journal.pone.0043764

**Published:** 2012-08-31

**Authors:** Olga Horakova, Dasa Medrikova, Evert M. van Schothorst, Annelies Bunschoten, Pavel Flachs, Vladimir Kus, Ondrej Kuda, Kristina Bardova, Petra Janovska, Michal Hensler, Martin Rossmeisl, Rui Wang-Sattler, Cornelia Prehn, Jerzy Adamski, Thomas Illig, Jaap Keijer, Jan Kopecky

**Affiliations:** 1 Department of Adipose Tissue Biology, Institute of Physiology Academy of Sciences of the Czech Republic v.v.i., Prague, Czech Republic; 2 Department of Human and Animal Physiology, Wageningen University, Wageningen, The Netherlands; 3 Research Unit of Molecular Epidemiology, Helmholtz Zentrum München, Neuherberg, Germany; 4 Institute of Experimental Genetics, Genome Analysis Center, Helmholtz Zentrum München, Neuherberg, Germany; Institut Pluridisciplinaire Hubert Curien, France

## Abstract

Insulin resistance, the key defect in type 2 diabetes (T2D), is associated with a low capacity to adapt fuel oxidation to fuel availability, i.e., metabolic inflexibility. This, in turn, contributes to a further damage of insulin signaling. Effectiveness of T2D treatment depends in large part on the improvement of insulin sensitivity and metabolic adaptability of the muscle, the main site of whole-body glucose utilization. We have shown previously in mice fed an obesogenic high-fat diet that a combined use of *n*-3 long-chain polyunsaturated fatty acids (*n*-3 LC-PUFA) and thiazolidinediones (TZDs), anti-diabetic drugs, preserved metabolic health and synergistically improved muscle insulin sensitivity. We investigated here whether *n*-3 LC-PUFA could elicit additive beneficial effects on metabolic flexibility when combined with a TZD drug rosiglitazone. Adult male C57BL/6N mice were fed an obesogenic corn oil–based high-fat diet (cHF) for 8 weeks, or randomly assigned to various interventions: cHF with *n*-3 LC-PUFA concentrate replacing 15% of dietary lipids (cHF+F), cHF with 10 mg rosiglitazone/kg diet (cHF+ROSI), cHF+F+ROSI, or chow-fed. Indirect calorimetry demonstrated superior preservation of metabolic flexibility to carbohydrates in response to the combined intervention. Metabolomic and gene expression analyses in the muscle suggested distinct and complementary effects of the interventions, with *n*-3 LC-PUFA supporting complete oxidation of fatty acids in mitochondria and the combination with *n*-3 LC-PUFA and rosiglitazone augmenting insulin sensitivity by the modulation of branched-chain amino acid metabolism. These beneficial metabolic effects were associated with the activation of the switch between glycolytic and oxidative muscle fibers, especially in the cHF+F+ROSI mice. Our results further support the idea that the combined use of *n*-3 LC-PUFA and TZDs could improve the efficacy of the therapy of obese and diabetic patients.

## Introduction

Combined treatments with multiple mechanisms of action are required for a better handling of metabolic diseases associated with obesity [1]–[4]. Thus, dietary, lifestyle, and pharmacological interventions should all be considered in the therapy of patients with type 2 diabetes (**T2D**), the major metabolic disease triggered by obesity [5].

Naturally occurring *n*-3 long-chain polyunsaturated fatty acids (***n*-3 LC-PUFA**), namely eicosapentaenoic acid (**EPA**; 20∶5n-3) and docosahexaenoic acid (**DHA**; 22∶6n-3) are now regarded as healthy constituents of diets for diabetic patients [6]–[8]. These lipids, which are abundant in sea fish, act as hypolipidemics and augment the efficacy of the lipid-lowering drugs [4], and reduce cardiac events and decrease progression of atherosclerosis (reviewed in ref. [7], [9], [10]). Numerous animal studies demonstrated reduced accumulation of body fat in response to dietary *n*-3 LC-PUFA supplementation [11]–[18], especially when combined with calorie restriction [19], reflecting possibly reduced proliferation of fat cells [12], [20], and/or metabolic changes in the liver [15], [21], adipose tissue [13], [19], and intestine [22]. In contrast, only few randomized clinical trials demonstrated a reduction of adiposity after *n*-3 LC-PUFA supplementation [23]–[26], while other studies in humans could not reveal any anti-obesity effect of *n*-3 LC-PUFA [27], [28]. Moreover, in rodents, *n*-3 LC-PUFA prevented [14], [21], [29]–[31] and even reversed [14], [32] insulin resistance induced by high-fat feeding, while *n*-3 LC-PUFA had little effect on glycemic control and insulin sensitivity in diabetic patients [23], [33], [34].

Impairment of insulin sensitivity represents the key defect in T2D. It is associated with a low capacity to adapt fuel oxidation to fuel availability, i.e., metabolic inflexibility [35], [36]. This results in lower glucose oxidation during insulin-stimulated conditions and in relatively low activation of lipid catabolism when lipids represent the main metabolic fuel, which further support accumulation of ectopic fat and lipotoxicity with a deleterious effect on insulin signaling [35]. Recent studies based on metabolomics suggest that both incomplete mitochondrial fatty acid oxidation and abnormal metabolism of branched-chain amino acids (**BCAA**) [37], [38] could contribute to insulin resistance, especially in the context of high fat-feeding in rodents and/or obesity (reviewed in [39]). In turn, this novel mechanistic insight may help to develop causal and more effective treatment strategies for T2D patients.

In our previous studies, we sought to learn whether *n*-3 LC-PUFA could augment the effects of anti-diabetic drugs, namely thiazolidinediones (**TZDs**). Thus, using a model of dietary obese mice and euglycemic-hyperinsulinemic clamps to measure insulin sensitivity, we have demonstrated that the combined use of *n*-3 LC-PUFA and TZD rosiglitazone, both administered at a relatively low dose (a ‘combined intervention’), exerted synergistic effects in prevention as well as reversal of insulin resistance [14]. These effects reflected a synergistic improvement in muscle insulin sensitivity [14], depending possibly in part on the induction of adiponectin [14], [32]. The combined intervention also exerted additivity in the counteraction of both dyslipidemia [14], [32] and low-grade inflammation of adipose tissue [14]. Also pioglitazone, a TZD used currently in treatment of diabetic patients [40], prevented both dyslipidemia and impairment of glucose homeostasis more efficiently in the combination with *n*-3 LC-PUFA as compared with the single intervention [32]. In addition, rosiglitazone, at the low dose used [14], [32], but not pioglitazone [32], augmented the anti-obesity effect of *n*-3 LC-PUFA. Changes in plasma metabolome suggested that the anti-obesity effect of the combined intervention reflected induction of fatty acid β-oxidation [32].

Motivated by our findings revealing synergistic effect of the combined use of *n*-3 LC-PUFA and rosiglitazone on muscle insulin sensitivity in dietary obese mice (refs. [14], [32]; see also above), and by the fact that skeletal muscle is the main site of glucose uptake [35], we aimed to verify a hypothesis that improvement of metabolic flexibility is an important part of the beneficial effects of the combined intervention. We also sought to learn what are the mechanisms underlying the improvement of muscle insulin sensitivity in response to the combined intervention. To examine this, we applied our established treatment protocol to high-fat diet-fed mice [14], [32]. Indirect calorimetry results indicated superior preservation of metabolic flexibility to carbohydrates in response to the combined intervention. Moreover, metabolomic analysis as well as evaluation of gene expression in skeletal muscle revealed (i) partially distinct mechanisms of action of *n*-3 LC-PUFA and rosiglitazone, and (ii) additive activation of the switch between glycolytic and oxidative muscle fibers in response to the combined intervention.

## Results

### Assessment of Intervention

**Figure 1:**
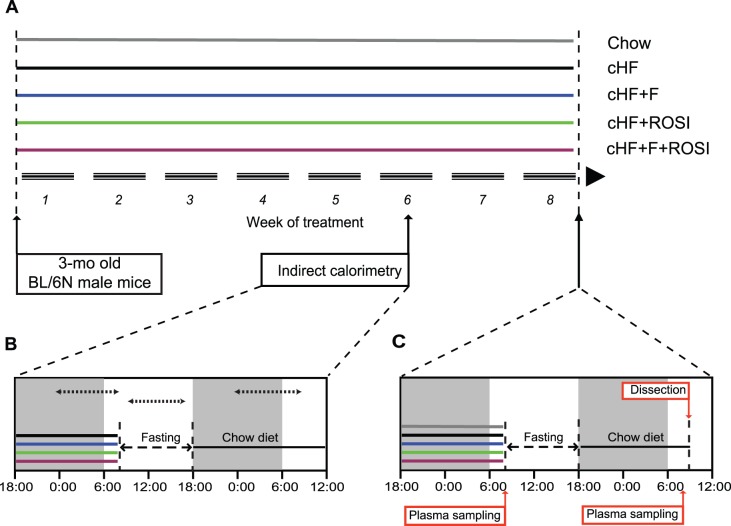
Overview of experimental setup. Starting at 3 months of age, subgroups of mice were fed either Chow or cHF diets, or subjected to various interventions (cHF+F, cHF+ROSI, and cHF+F+ROSI diets), which lasted for 8 weeks (**A**). During week 6 of the experiment, indirect calorimetry was performed using the ‘diet-switch protocol’ (**B**). At the end of the experiment, animals were killed either following the diet-switch protocol when re-fed Chow diet (**C**), or without any additional manipulations, while offered various cHF-based diets (not shown). White and grey background (**B, C**), light and dark phase of the day, respectively. Dotted arrow lines (**B**), periods of data collection for calculation of the mean values of *V*0_2_, RER, and PRCF (see Table 1 and Fig. 2).

In agreement with our previous experiments [14], [32], when adult mice (see Fig. 1A) were randomly assigned to an obesogenic high-fat diet (**cHF**) or to the following cHF-based diets supplemented with (i) *n*-3 LC-PUFA (**cHF+F)**; (ii) a low dose of rosiglitazone (**cHF+ROSI**); and (iii) both *n*-3 LC-PUFA concentrate and rosiglitazone (**cHF+F+ROSI)**, only the combined intervention prevented development of obesity during 8 weeks of the high-fat feeding experiment (Table 1). As observed before [14], [32], food consumption was not significantly affected by any of the interventions (data not shown).

**Table 1:** Growth characteristics and plasma parameters.

	Chow	cHF	cHF+F	cHF+ROSI	cHF+F+ROSI
Body weight (g)					
Initial	24.7±0.8	25.9±0.5	26.2±0.5	25.1±0.3	25.5±0.4
Final	31.4±1.0	37.6±1.1^f^	34.8±1.1	37.4±1.2^f^	32.3±0.8a,c
Body weight gain	5.7±0.5	11.7±1.3^f^	8.6±1.0	12.2±1.1^f^	6.8±0.9a,c
Plasma parameters					
cHF-based diets					
Triglycerides (mmol/l)	0.70±0.05	0.90±0.11	0.55±0.06	0.55±0.04^f^	0.46±0.03a,f
NEFA (mmol/l)	0.56±0.06	0.48±0.08	0.30±0.03^f^	0.44±0.05	0.33±0.05^f^
Glucose (mmol/l)	15.1±0.6	14.6±0.4	14.1±0.3	14.6±0.6	14.1±0.4
Insulin (nmol/l)	0.13±0.01	0.38±0.07^f^	0.24±0.03	0.30±0.03	0.19±0.05^a^
Re-fed Chow					
Triglycerides (mmol/l)	0.69±0.13	0.84±0.17	0.63±0.06	0.47±0.06	0.56±0.10
NEFA (mmol/l)	0.39±0.11	0.33±0.03	0.46±0.06	0.35±0.04	0.27±0.07
Glucose (mmol/l)	18.3±1.11	15.5±1.1	13.4±0.8^f^	16.2±0.7	15.2±0.5^f^
Insulin (nmol/l)	0.11±0.02	0.15±0.02	0.12±0.03	0.16±0.03	0.09±0.01
β-HB (µmol/l)	46.0±7.5	95.4±18.1^f^	68.1±6.9^f^	94.3±11.1^f^	35.2±6.4a,c
* n*	6	8–10	8–10	8–10	8–10

Three-month-old mice were placed on various diets and killed 8 weeks thereafter. Plasma parameters were followed as described in Methods, either in mice with free access to various cHF-based diets, or when mice were re-fed Chow (using the diet-switch protocol; see also Fig. 1C). BHB, β-hydroxybutyrate in the animals re-fed Chow.

a Significantly different from cHF;

b significantly different from cHF+F;

c significantly different from cHF+ROSI;

d significantly different from cHF+F+ROSI (ANOVA).

f Significantly different from Chow (*t*-test).

To evaluate plasma parameters at the end of the experiment at week 8, a ‘diet-switch protocol’ was applied (see Fig 1 and Methods), similarly as in the case of indirect calorimetry, which was performed during week 6 (see below). Thus, mice, which were fed during the whole experiment various cHF-based diets, were fasted during the light phase of the day and re-fed standard low-fat (**Chow**) diet during the night. Plasma was collected and glycemia was measured in (i) mice with *ad libitum* access to various cHF-based diets, before fasting, and (ii) mice re-fed Chow overnight. In the mice before fasting, the combined intervention strongly reduced plasma levels of triglycerides, even when compared with the mice fed Chow diet. Moreover, both *n*-3 LC-PUFA containing diets (cHF+F and cHF+F+ROSI) decreased NEFA levels in plasma (Table 1). Glycemia was not affected by any of the interventions (Table 1), in spite of the synergistic improvement of muscle insulin sensitivity in response to the combined intervention observed already before [14], [32]. Feeding cHF diet increased plasma insulin levels as compared with the Chow mice, while the combined intervention counteracted this effect (see also refs [14], [32]).

In the animals fasted during the day and re-fed Chow overnight, the hypolipidemic effects of *n*-3 LC-PUFA containing diets disappeared, and insulin levels in all the groups were similar to the Chow-fed control mice. Glycemia was similar in all the animals maintained on various cHF-based diets during the experiment, while insulin levels tended to be lower in the cHF+F+ROSI as compared with the cHF mice (Table 1). In addition, the combination intervention prevented cHF-induced elevation of β-hydroxybutyrate levels in plasma, while the single interventions had no effect (Table 1).

### Assessment of Metabolic Flexibility

**Figure 2:**
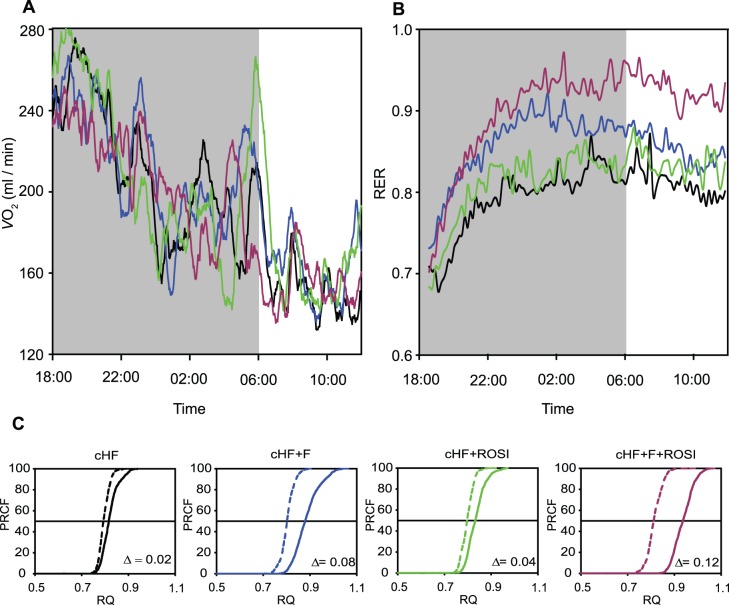
Indirect calorimetry. During week 6 of the experiment, in mice fed cHF diet , or mice subjected to various interventions (cHF+F, cHF+ROSI, and cHF+F+ROSI diets; *n* = 5; mice randomly chosen from each subgroup, see Table 1), indirect calorimetry was performed using the diet-switch protocol (Fig. 1B). Thus, during the first part of the measurements (between 6.00 p.m. and 8.00 a.m.), animals had *ad libitum* access to water and various cHF-based diets. After that period, the diets were removed and the animals fasted for 10 hours (between 8.00 a.m. and 6.00 p.m.). At the beginning of the dark phase of the day cycle (at 6.00 p.m.), all the subgroups were offered Chow diet and the measurements continued for 20 more hours. **A** and **B**. Oxygen consumption (**A**) and RER values (**B**) during re-feeding Chow diet (mean values). **C**. Plots of PRCF of RER values during the periods of fasting (broken lines; data collected between 9.00 a.m. and 5.00 p.m.) and re-feeding Chow (solid lines; data collected between 0.00 p.m. and 8.00 a.m.). Each curve represents the data pooled from all mice within a given group (*n* = 5; see above; ∼1,200 RER measurements per curve). For the means over different periods of the measurements and for the statistical analysis of these data, see Table 2.

During week 6 of the experiment, indirect calorimetry was performed, using the diet-switch protocol mentioned above, i.e., during the course of the subsequent periods of (i) feeding various cHF-based diets, (ii) fasting, and (iii) re-feeding Chow diet (i.e., low-fat diet with a carbohydrate/fat ratio of 16.3, wt/wt, as compared with the corresponding ratio of 1.0 in the case of the cHF-based diets; see Methods and ref. [14]). Neither oxygen consumption (***V*** **O_2_**), nor respiratory exchange ratio (**RER;** calculated as carbon dioxide production, ***V*** **CO_2_**, divided by *V*O_2_), the marker of fuel partitioning, were affected by any of the interventions, except for (i) a slight depression of *V*O_2_ in the cHF+F+ROSI mice during fasting (Table 2), and (ii) an increase in RER during re-feeding Chow diet in response to all the interventions. This increase was similar in the cHF and cHF+ROSI mice (Fig. 2B, Table 2), while the cHF+F mice exhibited a transiently higher induction during the second half of the dark phase of the day (Fig. 2B). Mice subjected to the combined intervention displayed the highest induction, which prevailed even during the light phase of the day (Fig. 2B). Percent relative cumulative frequency (**PRCF**) curves were constructed, based on RER values pooled from all animals within a given dietary group. This was done for the fasting and Chow periods (Fig. 2C). The values of log EC_50_ of PRCF (50^th^ percentile value) correspond to median RER values [41]. This robust approach confirmed the RER data, indicating a shift from lipid to carbohydrate oxidation in response to re-feeding mice Chow diet, with the highest flexibility in the mice subjected to the combined intervention (Fig. 2C and Table 2).

**Table 2:** Indirect calorimetry.

	cHF	cHF+F	cHF+ROSI	cHF+F+ROSI
*V*O_2_ (ml/min)				
Original diets	2.00±0.04	1.99±0.09	1.97±0.04	1.97±0.06
Fasting	1.83±0.06	1.83±0.03	1.94±0.08	1.69±0.04^b^
Re-feeding Chow	1.85±0.06	1.90±0.06	1.86±0.06	1.81±0.04
RER				
Original diets	0.84±0.01	0.84±0.01	0.83±0.01	0.87±0.01
Fasting	0.80±0.01	0.80±0.01	0.80±0.01	0.81±0.01
Re-feeding Chow	0.85±0.03	0.89±0.02	0.84±0.02	0.94±0.02^ab^
ΔRER	0.05±0.02	0.08±0.01	0.04±0.01	0.12±0.01^ab^

At 6 weeks after the initiation of the experiment, oxygen consumption (*V*O_2_) and carbon dioxide production were recorded every 2 min using indirect calorimetry. The measurements were performed following the diet-switch protocol in individual mice (Fig. 1B). During the first part of the measurements (between 6.00 p.m. and 8.00 a.m.), animals had *ad libitum* access to water and various cHF-based diets. After that period, the animals were fasted for 10 hours. At the beginning of the dark cycle at 6.00 p.m., all subgroups were switched to Chow diet, and the measurements continued for 20 more hours (‘Re-feeding Chow ‘). The measurements were performed under the 12-hour light-dark cycle (lights on from 6∶00 a.m.) at ambient temperature of 22°C. Data are means±SE (*n* = 5; mice randomly chosen from each subgroup, see Table 1) expressed for the following three time-periods (i) from 0.00 p.m. to 8.00 a.m., feeding various cHF-based diets; (ii) from 9.00 a.m. to 5 p.m., fasting; and (iii) from 0.00 p.m. to 8.00 a.m., re-feeding Chow. ΔRER, the difference in RER between mice re-fed Chow diet and fasted mice.

a Significantly different from cHF diet;

b significantly different from cHF+ROSI diet (ANOVA).

### Targeted Metabolomic Analysis in Skeletal Muscle

To characterize the role of muscle, the major organ of energy utilization, in the differential modulation of metabolic flexibility by the treatments, targeted metabolomics analysis in the muscle was performed in mice re-fed Chow diet (see also Fig. 1). Concentrations of 163 metabolites (Table S1) providing sets of hexoses, amino acids, sugars, acylcarnitines and phospholipids were measured using flow injection analysis/thermospray mass spectrometry (**FIA-MS**) with Biocrates Absolute*IDQ*™ targeted metabolomics technology.

**Figure 3:**
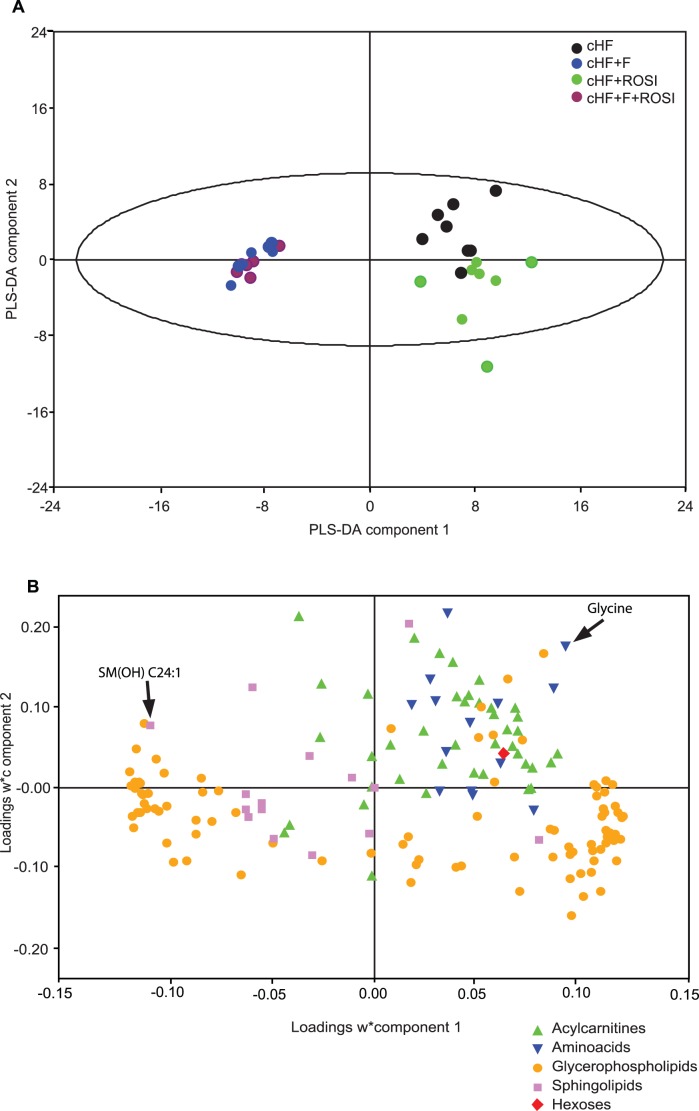
The effects of various interventions on gastrocnemius muscle metabolome. At 3 month of age, subgroups of mice were fed cHF diet, or subjected to various interventions using cHF-based diets (cHF+F, cHF+ROSI, and cHF+F+ROSI). Animals were killed while re-fed Chow diet (see the diet-switch protocol and Fig. 1). Targeted metabolomics analysis was performed in gastrocnemius muscle extracts. In total, concentrations of 163 metabolites were determined using FIA-MS with the Biocrates AbsoluteIDQ™ technology (see Table S1) and PLS-DA was performed. **A**. 2D-score scatter plot of the first (X-axis) and the second (Y-axis) PLS-DA component are shown for selected groups of mice (*n* = 7–8; mice randomly chosen from each subgroup, see Table 1). Mice were fed cHF (black circles), cHF+ROSI (green circles), cHF+F (blue circles), or cHF+F+ROSI (violet circles) diets. **B**. Corresponding loading scatter plot. Acylcarnitines (green triangle), amino acids (inverted blue triangle), glycerophospholipids (yellow circles), sphingolipids (violet circles) and sum of hexoses (red diamante) are shown. The score (**A**) and loading (**B)** plots complement each other. The position of objects (muscle sample) in a given direction in the score plot is determined by variables (metabolites) lying in same direction in the loading plot. For identification of the individual metabolites shown in **B**, see Fig. S1.

Partial least squares-discriminant analysis (**PLS-DA**) of the data separated mice into three distinct groups, namely the cHF mice, the cHF+ROSI mice, and the group of mice fed the diets containing *n*-3 LC-PUFA (both cHF+F and the cHF+F+ROSI mice; Fig. 3A). The first PLS-DA component (X-axis) showed a strong separation between the mice fed diets containing or not *n-3* LC PUFA, while the second PLS-DA component (Y-axis) showed a separation between the cHF mice and the cHF+ROSI mice. A loading scatter plot was constructed to determine the variables (metabolites) discriminating between the groups (Fig. 3B). Concerning the PLS-DA component 1, the most influential metabolites were glycerophospholipids, reflecting a difference in fatty acid composition of the diets. Most of sphingolipids were associated with the cHF+F and cHF+F+ROSI mice (Fig. 3B and Fig. S1). The metabolites having the greatest influence on the separation of the cHF mice were acylcarnitines and amino acids (Fig. 3B and Fig. S1).

**Figure 4:**
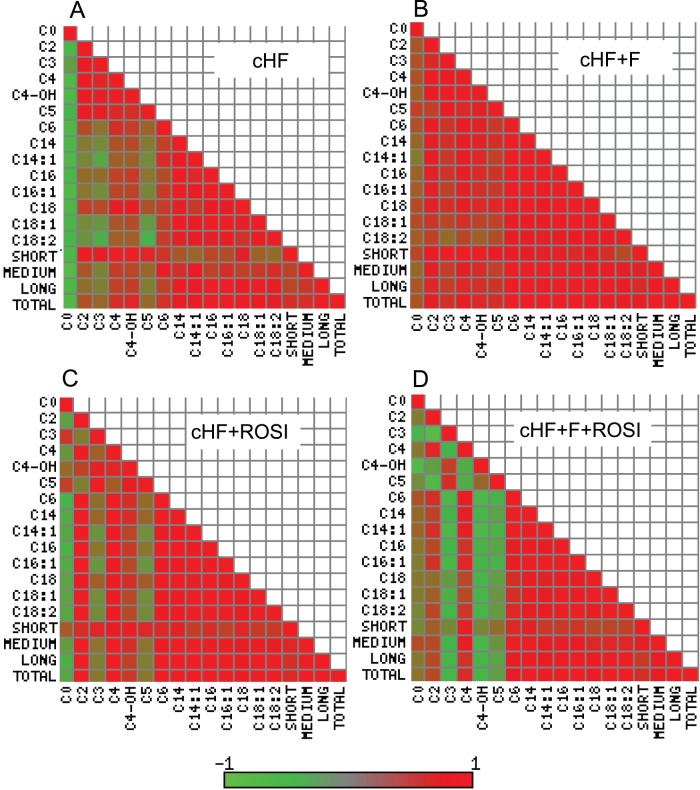
Heatmap analysis of the effects of various interventions on selected analytes in the muscle. Analysis was performed in mice re-fed Chow diet (see Fig. 1 and Fig. 3). Heatmap representations of the pairwise correlation matrix were generated using selected muscle metabolites in mice fed cHF (**A**), cHF+F (**B**), cHF+ROSI (**C**), and cHF+F+ROSI (**D**) diets. Each square represents Pearson correlation coefficient between the metabolite in the row with that in the column. The strength of correlation (red, positive; green, negative) is expressed as a color intensity, see the color scale bar. CX, acylcarnitine with the chain of X carbons; SHORT, sum of acylcarnitines C3-C7; MEDIUM, sum of carnitines C8-C13; LONG, sum of acylcarnitines C14–18; TOTAL, sum of all acylcarnitines.

**Figure 5:**
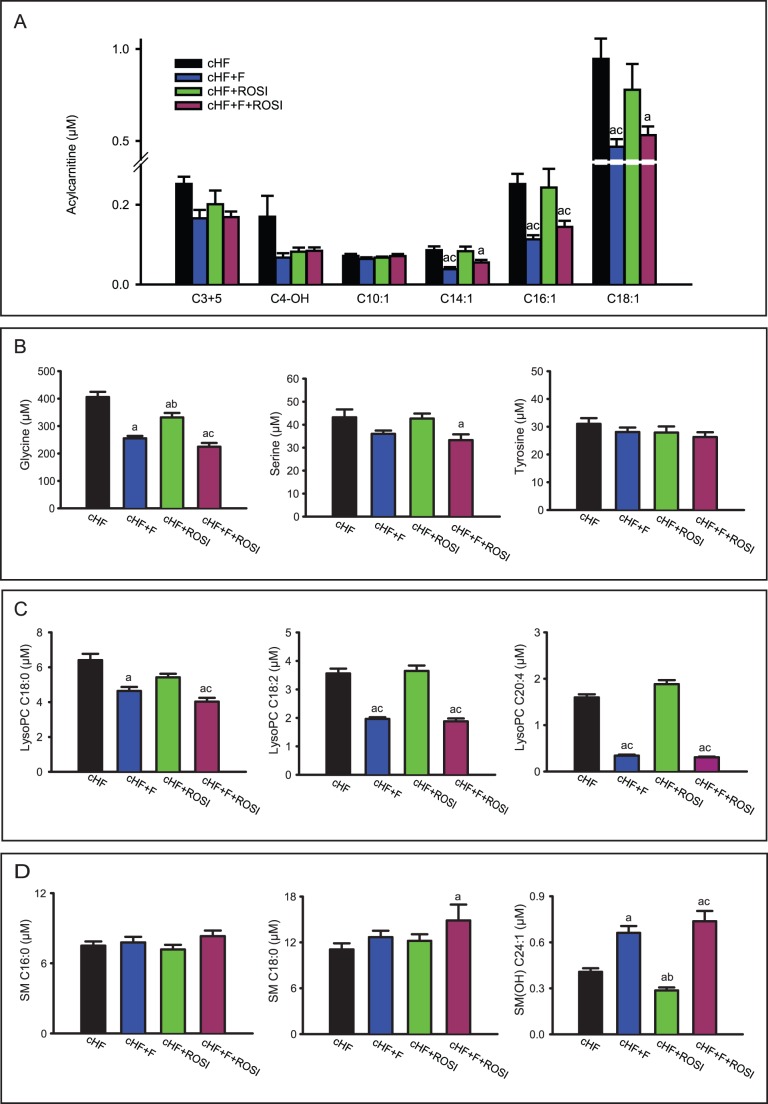
Concentrations of selected metabolites in gastrocnemius muscle extracts. Analysis was performed in mice re-fed Chow diet (see Fig. 3 and Table 1). **A.** Carnitines: propionyl-L-carnitine and isovalerlycarnitine (C3+C5); malonyl-L-carnitine (C4-OH); and various even-chain monounsaturated acylcarnitines (C10∶1, C14∶1, C16∶1, and C18∶1; individual acylcarnitines are denoted by their side chain; see Table S1). **B**. Amino acids. **C.** Lysophosphatidylcholines: stearoyl lysophasphatidylcholine (lysoPC C18∶0); linoleoyl lysophosphatidylcholine (lysoPC C18∶2); and arachidonoyl lysophasphatidylcholine (lysoPC C20∶4). **D.** Sphingolipids: palmitoyl sphingomyeline (SM C16∶0); stearoyl sphingomyeline (SM C18∶0); and hydroxysphingomyeline [SM(OH) C24∶1]. **D.** Data are means±SE (*n* = 7–8). Dietary groups are: cHF (black bars), cHF+F (blue bars); cHF+ROSI (green bars) and cHF+F+ROSI (violet bars). ^a^Significantly different from cHF; ^b^significantly different from cHF+F; ^c^significantly different from cHF+ROSI (ANOVA).

We generated heat maps of the correlation matrices of all pairwise correlations of muscle acylcarnitines (Fig. 4). These metabolites are formed in mitochondria, equilibrate with their cognate acyl CoAs and provide a detailed signature of mitochondrial fatty acid metabolism. Obesity accelerates fatty acid metabolism with possible accumulation of incomplete oxidation products, which may exacerbate insulin resistance [37], [38], [42]. Strong associations between all 14 acylcarnitines reflect complete β-oxidation of fatty acids, while reciprocal association between short-chain and long-chain acylcarnitines reflect a metabolic block, when fatty acids are metabolised only partially [37], [42]. Furthermore, acetylcarnitine (**C2**) can be used as a marker of acetyl-CoA levels, whereas odd-chain acylcarnitines, namely propionyl-L-carnitine (**C3**) and isovalerlycarnitine (**C5**), are primarily derived from catabolism of BCAA [37]–[39]. Our analysis revealed several hotspots and patterns, which discriminated between the interventions. The interpretations given above indicate that *n*-3 LC PUFA improved efficiency of β-oxidation (Fig. 4A vs Fig. 4B), while rosiglitazone alone had only a negligible effect (Fig. 4A vs Fig. 4C). The combined intervention resulted in a strong regulation of the metabolism of BCAA (C3 and C5 hotspots) and specifically unmasked the involvement of the hydroxylated C4 metabolite (**C4-OH**; Fig. 4D), i.e., either hydroxybutyrylcarnitine or malonylcarnitine (Table S1). This was supported by analysis at the level of individual metabolites, which revealed that even side-chain (C>10) acylcarnitines, arising from incomplete mitochondrial β-oxidation [37], [42], were significantly reduced in response to the *n*-3 LC-PUFA, while they were not affected by rosiglitazone. Levels of shorter side-chain (C<10) acylcarnitines did not change, while levels of odd-chain acylcarnitines (C3+C5) tended to be decreased by all the interventions. Together with the heatmap analysis, these results suggest different mechanisms engaged in the beneficial effects of the single interventions on insulin sensitivity. Thus, *n*-3 LC-PUFA ameliorate lipid-induced mitochondrial stress [37], [42], while the combination with *n*-3 LC-PUFA and rosiglitazone probably augments insulin sensitivity by the modulation of branched-chain amino acid metabolism [37], [38]. Furthermore, C4-OH levels were decreased by all the interventions (Table S1 and Fig. 5A) and *n*-3 LC-PUFA, especially in combination with rosiglitazone, exerted a suppressive effect on the concentration of free amino acids in the muscle (Table S1 and Fig. 5B). Glucogenic amino acids, namely glycine and serine, were more affected than amino acids that are both glucogenic and ketogenic, like tyrosine (Fig. 5B). These results are in favour of the synergistic improvement of muscle insulin sensitivity by the combined intervention (see Discussion).

In accordance with the changes in their plasma levels and amelioration of obesity-associated low-grade inflammation [32], the content of most of lysophosphatidylcholines (**lysoPCs**) were reduced in response to *n*-3 LC-PUFA also in the muscle, specifically lysoPCs with polyunsaturated fatty acids C18∶2-, C20∶3-, and C20∶4- side chains (Table S1 and Fig. 5C). The combined intervention tended to exert the most pronounced effects. Levels of sphingolipids (**SM**) were hardly regulated, except for hydroxysphingomyeline C24∶1 [**SM(OH) C24∶1**], which was strongly induced by *n*-3 LC-PUFA, especially in the combined intervention, while rosiglitazone alone had the opposite effect (Table S1 and Fig. 4D).

### Gene Expression in Skeletal Muscle

To investigate the effects of the interventions on gene expression in the skeletal muscle, whole genome microarray analysis was performed in mice re-fed Chow diet. As the initial step, three groups were compared, being the cHF+F and cHF+ROSI versus the control cHF mice. Non-stringent analysis of the results suggested that both single interventions regulated genes engaged in various biological processes, with only a partial overlap, and with a stronger effect of *n*-3 LC PUFA (Table 3). The stronger effect of *n*-3 LC PUFA agrees with the stronger effects observed for metabolomic data as analyzed by PLS-DA (Fig. 3A).

To examine the most prominent changes, genes with a significant regulation with an absolute fold change (**FC**) ≥1.5 were selected (Table S3). In total, 18 and 12 well annotated genes were detected when the effects of *n*-3 LC PUFA (Table S2; 12 genes down-regulated, 6 genes up-regulated in the cHF+F mice), and rosiglitazone (Table S3; 5 genes down-regulated, 7 genes up-regulated in the cHF+ROSI mice), respectively, were compared. All detected genes were unique, and mostly specific for the different interventions, while their number was very small compared to the number of genes detectable using the Agilent oligonucleotide arrays (see Methods).

**Table 3:** Differentially expressed, unique genes classified in biological processes.

	Upregulated		Downregulated		Total	
Single interventions	cHF+F	cHF+ROSI	cHF+F	cHF+ROSI	cHF+F	cHF+ROSI
Immune response	17	1	24	4	41	5
Development	15	5	12	2	27	7
G-protein signaling	9	0	13	0	22	0
Angiogenesis	11	4	10	8	21	12
Cell cycle	3	3	15	7	18	10
Transport	4	0	4	0	8	0
Total:	27	7	42	15	69	22

Combined intervention	Upregulated	Downregulated	Total			
Development	29	21	50			
Muscle contraction	8	18	26			
Apoptosis and survival	10	7	17			
Cell adhesion	2	12	14			
Angiogenesis	5	9	14			
Immune response	4	10	14			
Transcription	6	7	13			
G protein signaling	5	7	12			
Regulation of lipid metabolism	3	7	10			
Trafficking	5	1	6			
Total:	77	99	176			

Single interventions, unique genes with absolute FC ≥1.2 significantly different from cHF; combined intervention, unique genes with absolute FC ≥1.2 significantly different from cHF+F (*t*-test). Genes were manually classified in biological processes using scientific literature and bioinformatical resources, following initial MetaCore pathway analysis.

As the second step, we sought to find the genes, which could be additively/synergistically affected by the combined intervention by direct comparison of the cHF+F+ROSI versus cHF+ROSI mice. A higher number of genes was regulated as compared with the sum of genes regulated by single interventions (Table 3). Focusing on the most regulated genes using FC ≥1.5 as a threshold again, in total 39 well-annotated genes were identified to be differentially expressed, with most of these showing a lower expression in the cHF+F+ROSI mice (Table S4). The total number of differentially expressed genes, as well as the extent of their regulation was still relatively small (4 genes detected with FC ≥2.0; Table S4).

Detailed inspection of the expression data (Tables S2, S3, and S4) uncovered 10 genes, which were highly relevant with respect to a functional interpretation. Therefore, expression of these genes was verified using real-time quantitative RT-PCR (**qRT-PCR)** analysis across all the dietary groups. In addition, 5 genes, which were not identified using the microarrays, but could help the functional interpretation, were also included (for the complete list of genes, see Tables S5). Since the main focus of the analyses was on the characterisation of the muscle involvement in the differential effects of the interventions on metabolic flexibility, gene expression was evaluated not only in the animals re-fed Chow diet (see above), but also in mice from a separate experiment, which were killed when fed the cHF-based diets (see Methods and Fig. 1).

**Figure 6:**
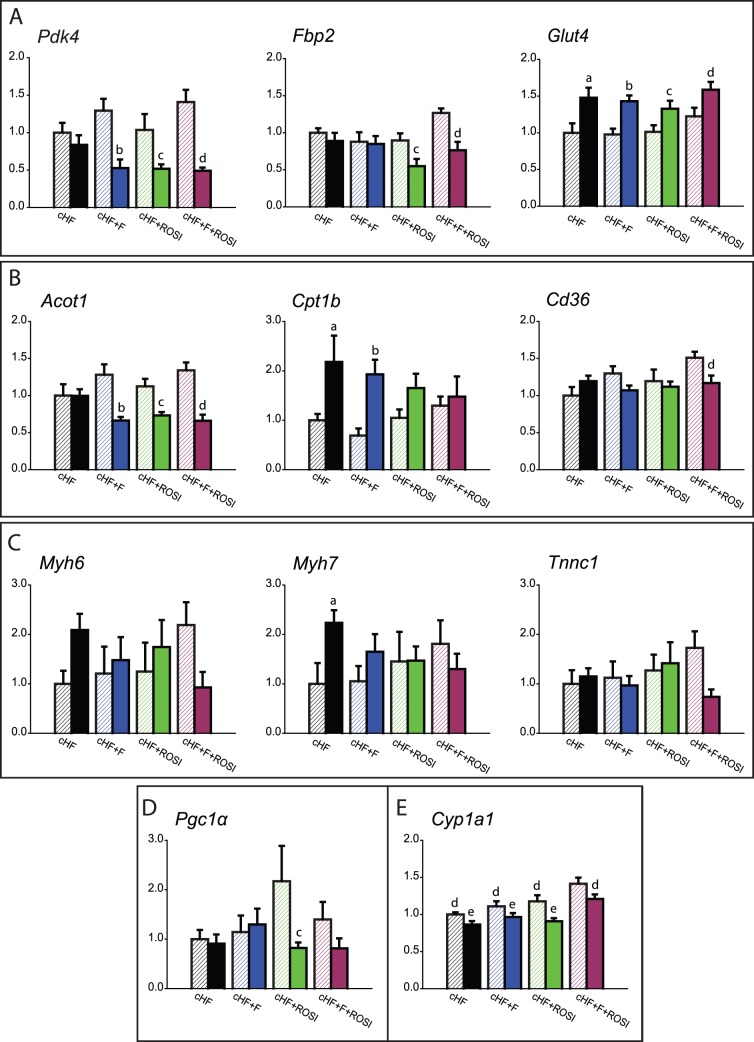
Expression of selected genes in gastrocnemius muscle. Mice were killed either without any additional manipulations, that is, while offered various ‘original’ cHF-based diets (**OrD**; crossed bars), or following the diet-switch protocol when re-fed Chow diet (full bars); see Fig. 1. **A**.Genes involved in carbohydrate metabolism: pyruvate dehydrogenase kinase isozyme 4 (*Pdk4*); fructose-1,6-bisphosphatase isoenzyme 2 (*Fbp2*); and glucose transporter type 4 (*Glut4*). **B**. Genes involved in lipid metabolism: acyl-CoA thioesterase 1 (*Acot1*); carnitine palmitoyltransferase 1b (*Cpt1b*); and CD36 antigen (*Cd36*). **C**. Slow muscle (oxidative) fiber genes: myosin, heavy polypeptide 6 (*Myh6*); myosin, heavy polypeptide 7 (*Myh7*); and troponin C type 1 (*Tnnc1*). **D.** *Pgc1α*. **E.** Cytochrome P450, family 1, subfamily a, polypeptide 1 (*Cyp1a1*). Data are means±SE (*n* = 7–8). See also Table S6. ^a^Significantly different from cHF, OrD; ^b^significantly different from cHF+F, OrD; ^c^significantly different from cHF+ROSI, OrD; ^d^significantly different from cHF+F+ROSI, OrD; ^e^significantly different from cHF+F+ROSI, re-fed Chow (ANOVA).

**Figure 7:**
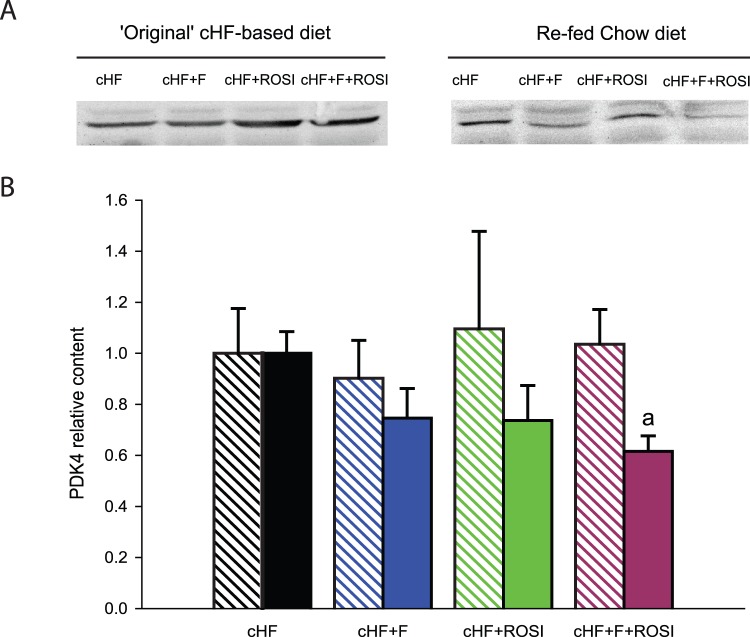
Content of PDK4 protein in gastrocnemius muscle. Mice were killed either without any additional manipulations, that is, while offered the ‘original’ cHF-based diet in fed state (crossed bars), or following the diet-switch protocol when re-fed Chow diet (full bars). **A**. Representative Western blot analysis. **B**. Quantification of PDK4 protein in skeletal muscle. Values are means±S.E. (*n* = 5–8).^ a^Significantly different from mice offered the cHF+F+ROSI diet (ANOVA).

Several genes involved in carbohydrate metabolism showed significant changes in expression (Fig. 6A): (i) pyruvate dehydrogenase kinase 4 (***Pdk4***), a regulatory enzyme limiting oxidation of glucose by inhibiting the pyruvate dehydrogenase complex [43], (ii) fructose-1,6-bisphophatase 2 (***Fbp2***), a key enzyme of gluconeogenesis catalyzing the hydrolysis of fructose 1,6-bisphosphate to fructose 6-phosphate and inorganic phosphate [44], and (iii) glucose transporter 4 (***Glut4***), which is essential for the insulin-stimulated glucose uptake in muscle cells [45]. Changes in *Pdk4* expression suggested improved metabolic flexibility in response to all the interventions, as demonstrated by the down-regulation of this gene in mice re-fed Chow diet (Fig. 6A), and they were verified using Western blot analysis (Fig. 7). In the case of *Fbp2*, only the rosiglitazone-based interventions could improved the flexibility, as revealed by the pronounced down-regulation of this gene in the mice re-fed Chow diet (Fig. 6A). Only in the case of *Glut4*, the combined intervention tended to increase the expression, independent on the actual feeding status (Fig. 6A).

Lipid metabolism was clearly regulated as well; genes encoding (i) acyl-CoA thioesterase 1 (***Acot1***), a mitochondrial enzyme hydrolyzing medium- and long-chain acyl-CoAs to the free fatty acid and CoASH [46], (ii) carnitine palmitoyltransferase 1b (muscle form; ***Cpt1b***), the rate-limiting transporter of activated fatty acids for mitochondrial β-oxidation in the muscle [47], and (iii) CD36 protein (***Cd36***), acting as a plasma membrane, TZD-inducible fatty acid transporter [48], were all regulated by the interventions (Fig. 6B). Both *Acot1* and *Cpt1b* showed a relatively strong response, while especially changes in *Cpt1b* expression suggested additive improvement of metabolic flexibility in response to the combined intervention. In contrast to the strong regulation of *Cpt1b* (Fig. 6B), expression of the liver isoform of the enzyme (***Cpt1a***) was not affected (Tables S5).

Furthermore, important changes were found in the expression of genes marking slow (oxidative) muscle fibres [49], [50], especially (i) myosin heavy polypeptides, ***Myh6*** and ***Myh7***, and (ii) troponin C 1 (***Tnnc1***; Fig. 6C). In the mice killed when fed the cHF-based diets, increased expression was observed for all these genes, suggesting additive/synergistic induction of the oxidative fibers, while in the mice re-fed Chow diet, expression of these genes was mostly down-regulated (*Myh6* and *Myh7*), with *Tnnc1* showing the strongest response to the combined intervention (Fig. 6C). The gene encoding peroxisome proliferator-activated receptor γ coactivator 1α, (***Pgc1α***), which stimulates the conversion of muscle fiber type towards oxidative type [51], [52], was regulated by rosiglitazone but not by *n*-3 LC PUFA (Fig. 6D).

Finally, among other genes showing a strong regulation, genes encoding enzymes involved in the production of lipid mediators from various PUFA [53], including a member of the cytochrome P450 family genes (***Cyp1a1***; Fig. 6E), were identified. In both cases, combined intervention resulted in the strongest induction, independent of the actual feeding status.

## Discussion

Indirect calorimetry performed in this study proved an additive improvement in metabolic flexibility in response to the combined use of *n*-3 LC-PUFA and rosiglitazone in mice fed an obesogenic high-fat diet. These results are in agreement with the changes of plasma metabolite levels during the fasted to re-fed transition, as well as with the synergistic improvement of muscle insulin sensitivity by the combined intervention in these animals [14], [32]. The beneficial effect on metabolic flexibility was observed during a switch from lipid to carbohydrate fuel. As reported recently by the group of Blaak, “the ability to switch from fat oxidation to carbohydrate oxidation after a meal is already impaired in the prediabetic state, suggesting this may be an early factor in the development toward type 2 diabetes” [54]. Interestingly, in our animals with *ad libitum* access to high-fat diet, no differences between the treatments in fuel partitioning were found, also in accordance with the lack of correlation between fasting RER and insulin sensitivity in some human studies (reviewed in [36]).

It is not known whether the effect of the combined intervention on metabolic flexibility merely reflects the reduced accumulation of body fat, when rosiglitazone is administered at a relatively low dose in the combination with *n*-3 LC-PUFA (see also [14], [32]), or whether it depends on direct effects on muscle metabolism. However, several pieces of evidence are in favor of the latter possibility. Thus, (i) in agreement with a previous study in rats [55], cHF+F diet, but not cHF+ROSI diet, increased RER values in mice re-fed Chow, in spite of the absence of an effect on body weight by neither of the single intervention; (ii) both metabolomic and gene expression analyses in the muscle documented several body weight-independent changes induced by the interventions (see below); and (iii) dietary supplementation with *n*-3 LC-PUFA, either in combination with rosiglitazone [14], [32] or pioglitazone [32] resulted in a synergistic induction of adiponectin, while elevated adiponectin levels were found to be associated with enhanced metabolic flexibility [56].

### Metabolic Profiling in the Muscle

Targeted metabolomic analysis in the muscle revealed relatively subtle differences between the interventions. This could be explained by the fact that the set of the measured metabolites lacked specific markers of carbohydrate metabolism, the process affected the most during the fasted to re-fed transition. Nevertheless, the levels of glucogenic amino acids were regulated as expected, with the strongest response induced by the combined intervention. Changes in the regulation and levels of acylcarnitines suggested distinct and complementary effects of *n*-3 LC-PUFA and rosiglitazone, with *n*-3 LC PUFA supporting mitochondrial β-oxidation (see [37], [38], [42]), and the combined intervention augmenting insulin sensitivity by the modulation of BCAA metabolism (see [37], [38]). In fact, in a previous study [57], the insulin-senzitizing potency of TZDs was shown to correlate with modulation of BCAA metabolism in WAT, with the impact on BCAA levels and insulin sensitivity in the muscle (see [39]). All the treatments decreased C4-OH, while its regulation with respect to the levels of other metabolites emerged as a unique marker of the effect of the combined intervention on muscle metabolome. This analyte could be either hydroxybutyrylcarnitine or malonylcarnitine, reflecting either the muscle levels of β-hydroxybutyrate and β-hydroxybutyryl-CoA [58], or α-hydroxybutyrate and α-hydroxybutyryl-CoA [59], or malonyl-CoA. All these metabolites could support muscle insulin sensitivity [14], [32] since (i) muscle β-hydroxybutyrate represents a strong marker and a possible causal factor for insulin resistance, which correlates with plasma NEFA levels [59]; (ii) α-hydroxybutyrate, a marker of mitochondrial redox status, is linked to the regulation of BCAA [59] and it was recently identified as an early biomarker of insulin resistance [60]; and (iii) malonyl-CoA is the key lipogenic intermediate controlling mitochondrial activity of β-oxidation by inhibiting CPT-1 (ref. [47]; see below).

Importantly, only the combined intervention could suppress plasma levels of β-hydroxybutyrate in mice re-fed Chow diet. In contrast, in the animals with *ad libitum* access to the cHF-based diets, or in fasted mice, no difference in the levels of β-hydroxybutyrate between was observed the interventions [32]. Formation of β-hydroxybutyrate occurs in the liver, as a by-product of β-oxidation when carbohydrates are scarce. Therefore, the marked suppression of the plasma β-hydroxybutyrate levels by the combined intervention suggests that, in addition to the beneficial muscle metabolic flexibility, also the adaptability of hepatic metabolism is synergistically improved. That this effect only occurred in the animals that switched to carbohydrate fuels supported the improvement in hepatic insulin sensitivity.

Chronic inflammation in obesity triggers insulin resistance [61], depending also on macrophage accumulation in the muscle and inflammatory state of muscle cells [62]. Therefore, we sought to detect changes in muscle metabolome, which could document anti-inflammatory effects of the treatments. Indeed, levels of several lysoPCs were decreased by the interventions, and the combined intervention tended to exert the strongest effect, in accordance with the notion that lysoPCs are associated with obesity-induced low-grade systemic inflammation (reviewed in [32]), and that phospholipase A2-derived lysoPC exert adverse effects on insulin responsiveness of myocytes [63].

Interestingly, we have found here a specific pattern of regulation of SM(OH) C24∶1 levels in the muscle, with the opposite effects by the single interventions, while combined intervention strongly increased the levels of this metabolite. As we have found before, SM(OH) C24∶1 represents the most strongly associated single metabolite with a genetic variant of serine-palmitoyltransferase [64], the rate-limiting enzyme in the synthesis of ceramides and sphingomyelins. Activity of this enzyme is increased in response to the treatments, which enhances insulin sensitivity, like aerobic training [65] or pioglitazone [66]. Moreover, the involvement of ceramide-derived sphingosines in the activation of AMP-activated protein kinase by adiponectin, downstream from the adiponectin receptors, was recently suggested [67]. This mechanism, which should operate in the muscle, suggests a new role for the sphingolipid metabolism [66] with respect to its long-disputed role in affecting muscle insulin sensitivity [65], [66], [68]–[70]. Our results further support the importance of SM(OH) C24∶1 as a marker of the muscle sphingosines metabolism.

### Gene Expression in the Muscle

Expression profiling in the muscle indicated a higher number of genes regulated in response to the combined intervention as compared with any of the single interventions, suggesting multiple mechanisms of action. Identification of the key metabolic genes was enabled by the quantitative comparison of the expression under the different metabolic states, i.e. in the animals relying mostly on either carbohydrate (mice re-fed Chow) or lipid fuels (mice with *ad libitum* access to the cHF-based diets). This approach revealed the involvement of both glycolysis and fatty acid oxidation in the preservation of the adaptability of muscle metabolism (see also ref. [71]); and moreover, it identified several candidates, namely *Pdk4*, *Fbp2* and *Cpt1b*, which could be involved in the additive effects of the combined intervention. That *Cpt1b* but not *Cpt1a* was regulated substantiates the view that *Cpt1b* was important for the modulation of fuel partitioning and β-oxidation by all the interventions, since it is primarily *Cpt1b* which is more sensitive to the inhibition by malonyl-CoA [47]. Previous studies suggested that oxidative (slow) muscle fibers could be important for metabolic flexibility [36], [49]. As compared with the glycolytic fibers, the oxidative fibers contain more mitochondria [72] and they are more insulin sensitive and more abundant in the muscle of insulin-sensitive than insulin-resistant human subjects [68]. Indeed, our unbiased analysis uncovered that several oxidative fibers genes, namely *Myh6, Myh7*, and *Tnnc1* were regulated in accordance with their involvement in metabolic flexibility. Our results suggest that the combined intervention potentiated the switch between the oxidative and glycolytic fibres, which is activated in response to the change of metabolic fuels. Thus, the combined intervention exhibited the most pronounced regulation of the oxidative fibres genes, while inducing their highest expression when the animals had *ad libitum* access to the cHF-based diets, and the lowest expression in the animals re-fed Chow. Interestingly, expression of *Pgc1α*, the master regulator of mitochondrial biogenesis and the marker of the slow muscle fibers [51], [52], matched the changes in the expression of the oxidative fibres genes only to a limited extent.

With respects to the anti-inflammatory effects of the interventions, meaningful changes in the expression of *Cyp1a1* were found, showing additive induction by the combination treatment. This gene is inducible by both TZDs [73], and PUFA [74], acts both as epoxygenase and hydroxylase, while producing PUFA-derived lipid mediators, with EPA-derived lipid mediators exerting presumably anti-inflammatory effects [53].

### Conclusions

**Figure 8:**
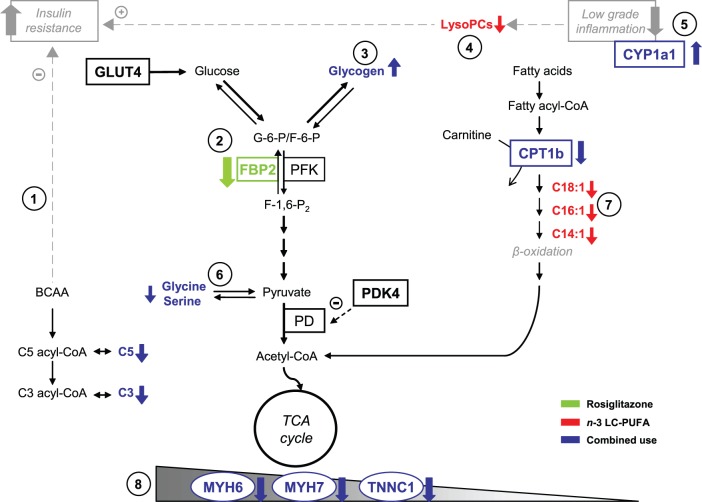
Synopsis of the results of metabolomic and gene expression analyses in the muscle. Metabolomic and gene expression analyses suggested complementary effects of the single interventions, with rosiglitazone augmenting insulin sensitivity by the modulation of branched-chain amino acid metabolism, especially when combined with *n*-3 LC-PUFA (1), and *n*-3 LC-PUFA supporting specifically complete oxidation of fatty acids in mitochondria (7). These beneficial metabolic effects were associated with inhibition of low grade tissue inflammation (5) and the activation of the switch between glycolytic and oxidative muscle fibers (8), especially in the combined intervention. Moreover, rosiglitazone inhibited gene expression of fructose-1,6-bisphosphatase 2 - a key enzyme of gluconeogenesis (2), while the concentrations of most of lysophosphatidylcholines were reduced in response to *n*-3 LC-PUFA (4). Glucogenic amino acids, namely glycine and serine, were affected by the combined intervention (6). As we published previously [14], the combined intervention also exerted synergistic stimulatory effect on muscle glycogen synthesis (3). Altered metabolites (bold font) and altered transcripts (bold font, rectangle) are marked. BCAA, branched-chain amino acids; C, acylcarnitine; CPT1b, carnitine palmitoyltransferase 1b; CYP1a1, member of the cytochrome P450 family genes; FBP2, fructose-1,6-bisphosphatase 2; GLUT4, glucose transporter 4; lysoPCs, lysophosphatidylcholines; PD, pyruvate dehydrogenase; PDK4 pyruvate dehydrogenase kinase 4; PFK, phosphofructokinase; MYH6 and MYH7, myosin heavy polypeptide; TCA cycle, tricarboxylic acid cycle; TNNC1, troponin C1.

Our results indicate that the combined use *n*-3 LC-PUFA and rosiglitazone preserve metabolic flexibility of mice fed an obesogenic high-fat diet, with a stronger, additive, effect as compared with any of the interventions applied individually (Fig. 8). Indirect calorimetry demonstrated that especially metabolic flexibility to carbohydrates was preserved in response to the combined intervention, also in agreement with the previously observed synergistic improvement of muscle insulin sensitivity under these conditions. Metabolomic and gene expression analyses suggested that both carbohydrate and lipid metabolism contribute to a better adaptability to fluctuating metabolic fuels and they highlighted the importance of mitochondrial fatty acid oxidation in the adaptability of muscle metabolism, while modulation of BCAA metabolism may be essential for the beneficial effect of the combined intervention on muscle insulin sensitivity. Our results further support the idea that dietary supplementation using *n*-3 LC-PUFA could improve the efficacy of TZDs, as well as other insulin-sensitizing and hypolipidemic pharmaceuticals used for the treatment of obese and type 2 diabetic patients.

## Methods

### Animals and Treatments

Male C57BL/6N mice (Charles River Laboratories, Sulzfeld, Germany) were maintained at 22°C on 12-h light-dark cycle (light from 6.00 a.m.) with free access to water and Chow diet (extruded Ssniff R/M-H diet; Ssniff Spezialdieten GmbH, Soest, Germany; with lipid, carbohydrate, and protein content ∼3.4, 55.3, and 19.3% wt/wt, respectively; energy density, 16.3 kJ/g). As described before [14], [32], three-month-old mice (Fig. 1) were randomly assigned (2 animals per cage) to cHF diet (lipid content ∼35% wt/wt, mainly corn oil; and carbohydrate, and protein content ∼35.4, and 20.5% wt/wt, respectively) or to the following ‘interventions’ using isocaloric cHF-based diets (energy density, 22.8 kJ/g), namely (i) cHF+F, cHF diet supplemented with *n*-3 LC-PUFA concentrate (46% DHA, 14% EPA, wt/wt, as triglycerides; product EPAX 1050 TG; EPAX a.s., Aalesund, Norway), which replaced 15% wt/wt of dietary lipids; (ii) cHF+ROSI, cHF diet supplemented with 10 mg rosiglitazone/kg diet (Avandia; GlaxoSmithKline, USA); and (iii) cHF+F+ROSI, cHF diet supplemented with both *n*-3 LC-PUFA concentrate and rosiglitazone. Chow diet-fed control mice were also included in the study. During the experiment lasting for 8 weeks (week 1– week 8), fresh ration of food was distributed daily and food consumption and body weights were recorded once a week. For the detailed fatty acid composition of lipids of all the diets, see [14].

Animals were killed by cervical dislocation under pentobarbital anesthesia (between 9.00 a.m. and 11.00 a.m.), (i) either when allowed free access to different cHF-based diets (*n* = 8), or (ii) using a ‘diet-switch protocol’ (Fig. 1) when the animals were first fasted for 10 hours during the light phase of the day (between 8.00 a.m. and 6.00 p.m.), than re-fed Chow (starting at 6.00 p.m.), and killed the following day (*n* = 10). In the second case, glycemia was evaluated and EDTA-plasma for measurement of various metabolites (see below) was collected in fed state, at 7.00 a.m., either before the initiation of the fasting period (when fed various cHF-based diets), or before the killing (when re-fed Chow; Fig. 1). In both cases, gastrocnemius muscle was dissected for RNA analysis (see below).

The animal experiments were specifically approved by the Animal Care and Use Committee of the Institute of Physiology Academy of Sciences of the Czech Republic v.v.i. (Approval Number: 172/2009) and conducted under the guidelines.

### Indirect Calorimetry

To evaluate energy expenditure, as well as metabolic flexibility to the high-carbohydrate meal, indirect calorimetry was performed using system INCA (Somedic, Horby, Sweden) [19], [41] during week 6 of the experiment (Fig. 1) in singly caged mice (Eurostandard type II mouse plastic cages, ~ 6,000 ml; Techniplast, Milan, Italy), which were placed in sealed measuring chambers equipped with thermostatically controlled heat exchangers at 22°C. *V*O_2_ and *V*CO_2_ were recorded every 2 min under a constant airflow rate (1000 ml/min). The diet-switch protocol (see above and Fig. 1) was used, allowing for the evaluation of metabolic flexibility, which was assessed as the induction in RER in response to the re-feeding Chow diet, following the period of fasting. Alternatively, to assess the change in fuel partitioning more accurately, PRCF curves were constructed based on RER values pooled from all animals within a given dietary group during the specific period of the measurement. Provided that PRCF curves represent the normally distributed data, the values of log EC_50_ of PRCF (50^th^ percentile value) correspond to RER values [41].

### Metabolite Quantification

NEFA, triglycerides, and β-hydroxybutyrate in EDTA-plasma and glycemia were assessed as before [32]. In addition, targeted metabolomics analysis was performed using extracts [75] from skeletal muscle (100 mg aliquots; samples collected in our previous study [32]) to determine concentrations of 163 metabolites using a targeted metabolomics kit (AbsoluteIDQTM kit p150, Biocrates Life Sciences AG, Innsbruck, Austria) based on FIA-MS as before [32], [64]. Concentrations of all analyzed metabolites are reported in µM. For the general information on biological roles of the metabolites, see [64]. In short, 14 amino acids, sum of hexoses, free carnitine, 26 acylcarnitines, 14 hydroxy- and dicarboxy-acylcarnitines, 10 sphingomyelins, 5 hydroxysphingomyelins, 38 diacyl-phosphatidylcholines, 39 acyl-alkyl-phospatidylcholines, and 15 lysophosphatidylcholines were quantified. For the full list of the measured metabolites and the abbreviations to denote them, see Table S1.

### Gene Expression in Skeletal Muscle

Total RNA was isolated from gastrocnemius muscle samples stored in RNA*later* (Ambion, Austin, TX, USA) at −20°C using TRI Reagent (Sigma-Aldrich, St. Louis, MO, USA) according to the manufacturer’s instruction. After extraction, RNA was purified by using RNeasy columns (Qiagen, Venlo, The Netherlands). RNA concentration and purity were measured using the NanoDrop spectrophotometer (IsoGen Life Science, Maarsen, The Netherlands). The integrity of RNA was checked with Experion automated electrophoresis system (BioRad, Veenendaal, The Netherlands). Levels of selected gene transcripts in total muscle RNA from individual mice were evaluated using real-time qRT-PCR [13] and appropriate primers (see Table S5).

Muscle total RNA isolated from individual mice was also analyzed using Agilent whole genome 44K mouse gene expression arrays (Agilent Technologies, Inc., Santa Clara, CA, USA), similarly as described before (ref. [76]; see Supporting Information S1). The arrays contain 43,379 probes (excluding controls). Differential gene expression was assessed in two subsequent microarray analyses using a reference pool design. In the first analysis mice (*n* = 7–8) of the cHF, cHF+F, and cHF+ROSI treatment groups were assessed and in the second analysis, the cHF+ROSI and cHF+F+ROSI were assessed. In total, after removing probes that were below two times the background, 20,721 probes remained in the first study and 25,922 probes in the second study. Quality control and data handling was done as described [76]. More detailed information, including data analysis, is provided in Supporting Information S1. The gene expression studies are deposited in Gene Expression Omnibus of NCBI with GEO accession number GSE36718.

### Western Blot Analysis of Protein Levels of PDK4

Frozen muscles (50 mg) were homogenized in 0.3 ml of ice-cold lysis buffer (20 mmol/l Tris, pH 7.4; 1 mmol/l EDTA; 1% deoxycholate; 1% Triton X-100; 1 mmol/l phenylmethylsulfonyl fluoride; 10 µg/ml aprotinin; 10 µg/ml leupeptin and Phosphatase-Inhibitor-Mix I [Serva, Heidelberg, Germany]). Tissue lysates were centrifuged at 15,000 g for 10 minutes at 4°C. Protein concentration in supernatant was determined by bicinchoninic acid assay. 50 µg protein was separated by 10% sodium dodecylsulphate-polyacrylamide gel electrophoresis and transferred to Immobilon-FL membranes (Millipore, Billerica, MA, USA). The membranes were then probed with rabbit antibodies against PDK4 (1∶200; Abgent, San Diego, CA, USA). Secondary antibody conjugated to IRDye 800CW (Li-COR, Lincoln, NE, USA) was used for detection. Membranes were scanned using an Odyssey IR scanner (LI-COR). The results were quantified using AIDA image analysis software (Raytest, Straubenhardt, Germany).

### Statistical Analysis

Growth characteristics, plasma parameters, indirect calorimetry data, and metabolite concentration profile data were analyzed using ANOVA and paired *t*-test as before [19], [32]. All values are presented as means±SE. Comparisons were judged to be significant at *p*≤0.05. Correlation matrices were computed using MS Excel. Gene array data was processed with MetaCore Pathway analysis software (GeneGo, Carlsbad, CA, USA). PLS-DA was performed using Umetrics SIMCA-P+12 statistical software (Umetrics AB, Umea, Sweden) as before [32]. When the PLS-DA score plot showed significantly separated groups, a loading scatter plot was constructed to determine the variables influencing their separation.

## Supporting Information

Figure S1 **The effects of various interventions on gastrocnemius muscle metabolome - detailed loading scatter plot**. Identification of the metabolites shown in the simplified loading scatter plot (Fig. 3B). Acylcarnitines (green triangle), aminoacids (inverted blue triangle), lysophosphatidylcholines (yellow circles), diacyl phosphatidylcholines (orange circles), acyl-alkyl phosphatidylcholines (red circles), sphingolipids (violet circles) and sum of hexoses (dark green diamante) are shown. Individual metabolites are indicated by unique numbers (see Table S1 for the name of each metabolite).
(EPS)

Table S1 **List of all 163 metabolites measured in skeletal muscle extracts - targeted metabolomics analysis**. In short, 14 amino acids, sum of hexoses (H1), free carnitine (C0), 26 acylcarnitines (C2, C3,…C18∶2), 14 hydroxy- and dicarboxy-acylcarnitines (C-3OH, C4-OH, C5-DC,…C18∶1-OH), 10 sphingomyelins (SM C16∶0, SM C16∶1,… SM C26∶1), 5 hydroxysphingomyelins [SM(OH)C14∶1… SM(OH)C24∶1], 38 diacyl-phosphatidylcholines (PC aa C24∶0 …PC aa C42∶6), 39 acyl-alkyl-phospatidylcholines (PC ae C30∶0… PC ae C44∶6), and 15 lyso-phosphatidylcholines (lysoPC a C6∶0….lysoPC a C28∶1) were identified in extracts of skeletal muscle from animals subjected to various interventions and killed when re-fed Chow (see Methods and Fig. 1). Concentrations of all analysed metabolites are reported in µM. All metabolites above were determined using flow injection analysis/thermospray mass spectrometry (**FIA-MS**) with Biocrates Absolute*IDQ*™ targeted metabolomics technology. Data are means±SE (*n* = 7–8).
(XLS)

Table S2 **Differentially regulated probesets expressed in cHF+F versus control cHF dietary groups.** The data provided represents only the statistical significant differentially expressed probesets of the microarrays (cHF: *n* = 8, cHF+F: *n* = 8) which showed a mean absolute fold change ≥1.5 (cHF+F/cHF).
(DOC)

Table S3 **Differentially regulated probesets expressed in cHF+ROSI versus cHF dietary groups.** The data provided represents only the statistical significant differentially expressed probesets of the microarrays (cHF+ROSI: *n* = 8, cHF: *n* = 8) which showed a mean absolute fold change ≥1.5 (cHF+ROSI/cHF).
(DOC)

Table S4 **Differentially regulated probesets expressed in cHF+F+ROSI versus cHF+ROSI dietary groups.** The data provided represents only the statistical significant differentially expressed probesets of the microarrays (cHF+F+ROSI: *n* = 8, cHF+ROSI: *n* = 8) which showed a mean absolute fold change ≥1.5 (cHF+F+ROSI/cHF+ROSI).
(DOC)

Table S5 **Real-time quantitative RT-PCR analysis: genes and primers.**
(DOC)

Table S6 **Real-time quantitative RT-PCR analysis in skeletal muscle.** At the end of the experiment, mice were killed either without any additional manipulations, that is, while offered the ‘original’ cHF-based diets (OrD), or following the ‘diet-switch protocol’ when re-fed Chow diet; see Fig. 1. Control mice maintained on Chow diet throughout the intervention and killed in *ad libitum* fed state were also analyzed. Data are means±SE (*n* = 7–8). For gene symbols, see Table S5. ^a ^Significantly different from cHF, OrD. ^b^ significantly different from cHF+F, OrD. ^c^ significantly different from cHF+ROSI, OrD. ^d^ significantly different from cHF+F+ROSI, OrD. ^e^ Significantly different from cHF, Re-fed Chow. ^f^ significantly different from cHF+F, Re-fed Chow. ^g ^significantly different from cHF+ROSI, Re-fed Chow. ^h ^significantly different from cHF+F+ROSI, Re-fed Chow (Two-way ANOVA). ^i ^significantly different from Chow (t-test).
(DOC)

Supporting Information S1 (DOC)
