# Supplementary material for: Preservation of Metabolic Flexibility in Skeletal Muscle by a Combined Use of n-3 PUFA and Rosiglitazone in Dietary Obese Mice
Source: PLoS One. 2012 Aug 31;7(8):e43764. doi: 10.1371/journal.pone.0043764 (PMC3432031; doi:10.1371/journal.pone.0043764)
Supplement: Table S2 — Differentially regulated probesets expressed in cHF+F versus control cHF dietary groups. The data provided represents only the statistical significant differentially expressed probesets of the microarrays (cHF: n = 8, cHF+F: n = 8) which showed a mean absolute fold change ≥1.5 (cHF+F/cHF). (DOC) [file pone.0043764.s003.doc]

**Table S2** Differentially regulated probesets expressed in cHF+F versus control cHF dietary groups

| **Probe name** | **Gene symbol** | **Description** | **Fold change** |
| --- | --- | --- | --- |
| **Down-regulated** | | | |
| A_52_P682382 | *Scd1* | stearoyl-Coenzyme A desaturase 1 | -2.04 |
| A_52_P262219 | *Fos* | FBJ osteosarcoma oncogene | -2.03 |
| A_52_P657360 | *Tnni1* | troponin I, skeletal, slow 1 | -1.79 |
| A_51_P143162 | *Myh7* | myosin, heavy polypeptide 7, cardiac muscle, beta | -1.77 |
| A_51_P512210 | *Myh6* | myosin, heavy polypeptide 6, cardiac muscle, alpha | -1.70 |
| A_51_P461894 | *Tnnc1* | troponin C, cardiac/slow skeletal | -1.65 |
| A_52_P362772 | *AK048349* | 16 days embryo head cDNA, RIKEN full-length enriched library, clone:C130051M16 product:SNF1-like kinase, full insert sequence | -1.62 |
| A_51_P189814 | *Cldn5* | claudin 5 | -1.61 |
| A_51_P293069 | *9630055N22Rik* | RIKEN cDNA 9630055N22 gene | -1.56 |
| A_51_P451574 | *Acot1* | acyl-CoA thioesterase 1 | -1.56 |
| A_52_P763309 | *AK033367* | 16 days embryo lung cDNA, RIKEN full-length enriched library, clone:8430406N14 product:unclassifiable, full insert sequence | -1.54 |
| A_51_P462153 | *Tpm3* | tropomyosin 3, gamma | -1.51 |
| **Up-regulated** | | | |
| A_52_P651784 | *LOC672434* | PREDICTED: similar to S-adenosylmethionine decarboxylase 1 | 1.55 |
| A_52_P1115511 | *6030422H21Rik* | 13 days embryo male testis cDNA, RIKEN full-length enriched library, clone:6030422H21 product:unclassifiable, full insert sequence | 1.59 |
| A_51_P331570 | *Trib3* | tribbles homolog 3 | 1.62 |
| A_52_P184149 | *Mthfd2* | methylenetetrahydrofolate dehydrogenase (NAD+ dependent), methenyltetrahydrofolate cyclohydrolase | 1.64 |
| A_52_P645855 | *D5Ertd593e* | DNA segment, Chr 5, ERATO Doi 593, expressed | 1.65 |
| A_51_P369762 | *Itgb1bp3* | integrin beta 1 binding protein 3 | 1.86 |

The data provided represents only the statistical significant differentially expressed probesets of the microarrays (cHF: *n*=8, cHF+F: *n*=8) which showed a mean absolute fold change ≥ 1.5 (cHF+F/cHF).
