# Supplementary material for: Preservation of Metabolic Flexibility in Skeletal Muscle by a Combined Use of n-3 PUFA and Rosiglitazone in Dietary Obese Mice
Source: PLoS One. 2012 Aug 31;7(8):e43764. doi: 10.1371/journal.pone.0043764 (PMC3432031; doi:10.1371/journal.pone.0043764)
Supplement: Table S3 — Differentially regulated probesets expressed in cHF+ROSI versus cHF dietary groups. The data provided represents only the statistical significant differentially expressed probesets of the microarrays (cHF+ROSI: n = 8, cHF: n = 8) which showed a mean absolute fold change ≥1.5 (cHF+ROSI/cHF). (DOC) [file pone.0043764.s004.doc]

**Table S3** Differentially regulated probesets expressed in cHF+ROSI versus cHF dietary groups

| **Probe name** | **Gene symbol** | **Description** | **Fold change** |
| --- | --- | --- | --- |
| **Down-regulated** | | | |
| A_51_P235945 | *Hp* | haptoglobin | -1.96 |
| A_51_P350453 | *Pdk4* | pyruvate dehydrogenase kinase, isoenzyme 4 | -1.88 |
| A_51_P109369 | *Fbxo32* | F-box protein 32 | -1.55 |
| A_52_P380379 | *Ucp3* | uncoupling protein 3 (mitochondrial, proton carrier) | -1.52 |
| A_51_P415395 | *3300001A09Rik* | RIKEN cDNA 3300001A09 gene | -1.51 |
| **Up-regulated** | | | |
| A_51_P389479 | *1190003J15Rik* | 18-day embryo whole body cDNA, RIKEN full-length enriched library, clone:1190003J15 product:hypothetical Transthyretin/Transthyretin-related containing protein, full insert sequence | 1.51 |
| A_52_P1179878 | *AK036012* | 16 days neonate cerebellum cDNA, RIKEN full-length enriched library, clone:9630027C04 product:unclassifiable, full insert sequence | 1.51 |
| A_52_P1115511 | *6030422H21Rik* | 13 days embryo male testis cDNA, RIKEN full-length enriched library, clone:6030422H21 product:unclassifiable, full insert sequence | 1.55 |
| A_51_P230269 | *H2-Q10* | histocompatibility 2, Q region locus 10 | 1.60 |
| A_51_P283456 | *Cyp2e1* | cytochrome P450, family 2, subfamily e, polypeptide 1 | 1.63 |
| A_51_P238576 | *Cyp4a14* | cytochrome P450, family 4, subfamily a, polypeptide 14 | 1.90 |
| A_52_P348256 | *Mup1* | major urinary protein 1 | 1.93 |

The data provided represents only the statistical significant differentially expressed probesets of the microarrays (cHF+ROSI: *n*=8, cHF: *n*=8) which showed a mean absolute fold change ≥ 1.5 (cHF+ROSI/cHF).
