# Supplementary material for: Preservation of Metabolic Flexibility in Skeletal Muscle by a Combined Use of n-3 PUFA and Rosiglitazone in Dietary Obese Mice
Source: PLoS One. 2012 Aug 31;7(8):e43764. doi: 10.1371/journal.pone.0043764 (PMC3432031; doi:10.1371/journal.pone.0043764)
Supplement: Table S4 — Differentially regulated probesets expressed in cHF+F+ROSI versus cHF+ROSI dietary groups. The data provided represents only the statistical significant differentially expressed probesets of the microarrays (cHF+F+ROSI: n = 8, cHF+ROSI: n = 8) which showed a mean absolute fold change ≥1.5 (cHF+F+ROSI/cHF+ROSI). (DOC) [file pone.0043764.s005.doc]

**Table S4** Differentially regulated probesets expressed in cHF+F+ROSI versus cHF+ROSI dietary groups

| **Probe name** | **Gene symbol** | **Description** | **Fold change** |
| --- | --- | --- | --- |
| **Down-regulated** | | | |
| A_51_P501844 | *Cyp26b1* | cytochrome P450, family 26, subfamily b, polypeptide 1 | -2.15 |
| A_51_P369426 | *Zmynd17* | 0 day neonate head cDNA, RIKEN full-length enriched library, clone:4833444M15 | -2.12 |
| A_51_P133684 | *Csrp3* | cysteine and glycine-rich protein 3 | -1.86 |
| A_52_P381484 | *Spon2* | spondin 2, extracellular matrix protein | -1.81 |
| A_52_P576720 | *Calm4* | calmodulin 4 | -1.80 |
| A_51_P324814 | *Krt18* | keratin 18 | -1.79 |
| A_52_P556140 | *Vapb* | vesicle-associated membrane protein, associated protein B and C | -1.78 |
| A_52_P408826 | *9530083O12Rik* | adult male urinary bladder cDNA, RIKEN full-length enriched library, clone:9530083O12 product:unclassifiable, full insert sequence. | -1.78 |
| A_52_P682382 | *Scd1* | stearoyl-Coenzyme A desaturase 1 | -1.78 |
| A_51_P194230 | *Zic1* | zinc finger protein of the cerebellum 1 | -1.74 |
| A_52_P622680 | *Chmp4b* | chromatin modifying protein 4B | -1.72 |
| A_52_P677718 | *Tatdn2* | TatD DNase domain containing 2 | -1.66 |
| A_51_P104418 | *Dusp10* | dual specificity phosphatase 10 | -1.65 |
| A_51_P111462 | *Arl15* | ADP-ribosylation factor-like 15 | -1.65 |
| A_52_P591431 | *Tra2a* | transformer 2 alpha homolog (Drosophila) | -1.61 |
| A_51_P235977 | *4933401F05Rik* | adult male testis cDNA, RIKEN full-length enriched library, clone:4933401F05 product:hypothetical Serine proteases, trypsin family containing protein, full insert sequence | -1.60 |
| A_52_P659312 | *Spsb4* | splA/ryanodine receptor domain and SOCS box containing 4 | -1.59 |
| A_51_P294535 | *Unc5b* | unc-5 homolog B (C. elegans) | -1.58 |
| A_51_P324450 | *Pbp2* | phosphatidylethanolamine binding protein 2 | -1.58 |
| A_52_P486964 | *NAP051367-1* | Unknown | -1.56 |
| A_51_P200529 | *Mpst* | mercaptopyruvate sulfurtransferase | -1.55 |
| A_51_P100505 | *AK054424* | 2 days pregnant adult female ovary cDNA, RIKEN full-length enriched library, clone:E330024G11 product:unclassifiable, full insert sequence. | -1.55 |
| A_51_P346445 | *Hspb7* | heat shock protein family, member 7 (cardiovascular) | -1.54 |
| A_51_P335000 | *Fhl1* | four and a half LIM domains 1 | -1.51 |
| A_51_P260265 | *Hoxd4* | homeo box D4 | -1.51 |
| A_51_P316243 | *C330024D12Rik* | ES cells cDNA, RIKEN full-length enriched library, clone:C330024D12 product:hypothetical Pyrrolidone carboxyl peptidase (pyroglutamate aminopeptidase) structure containing protein, full insert sequence. | -1.50 |
| **Up-regulated** | | | |
| A_51_P458130 | *Tph1* | tryptophan hydroxylase 1 | 1.51 |
| A_52_P430194 | *Smox* | 0 day neonate eyeball cDNA, RIKEN full-length enriched library, clone:E130115D16 product:POLYAMINE OXIDASE ISOFORM-3 homolog [Homo sapiens], full insert sequence. | 1.51 |
| A_51_P112734 | *Slc7a8* | solute carrier family 7 (cationic amino acid transporter, y+ system), member 8 | 1.52 |
| A_52_P1179878 | *AK036012* | 16 days neonate cerebellum cDNA, RIKEN full-length enriched library, clone:9630027C04 product:unclassifiable, full insert sequence. | 1.52 |
| A_51_P279693 | *Cyp1a1* | cytochrome P450, family 1, subfamily a, polypeptide 1 | 1.55 |
| A_51_P499020 | *Fbp2* | fructose bisphosphatase 2 | 1.56 |
| A_52_P706912 | *AK035307* | adult male urinary bladder cDNA, RIKEN full-length enriched library, clone:9530013H21 product:unclassifiable, full insert sequence. | 1.57 |
| A_52_P175376 | *Tcfcp2l1* | transcription factor CP2-like 1 | 1.57 |
| A_52_P355480 | *TC1677116* | Q4QRG7_BRARE (Q4QRG7) Zgc:113884, partial (10%) | 1.58 |
| A_52_P453884 | *Foxo1* | forkhead box O1 | 1.65 |
| A_52_P835711 | *AK043497* | 10 days neonate cortex cDNA, RIKEN full-length enriched library, clone:A830001K22 product:unclassifiable, full insert sequence. | 1.73 |
| A_51_P331570 | *Trib3* | tribbles homolog 3 (Drosophila) | 1.93 |
| A_51_P369762 | *Itgb1bp3* | integrin beta 1 binding protein 3 | 2.11 |
| A_52_P184149 | *Mthfd2* | methylenetetrahydrofolate dehydrogenase (NAD+ dependent), methenyltetrahydrofolate cyclohydrolase | 2.16 |

The data provided represents only the statistical significant differentially expressed probesets of the microarrays (cHF+F+ROSI: *n*=8, cHF+ROSI: *n*=8) which showed a mean absolute fold change ≥ 1.5 (cHF+F+ROSI/cHF+ROSI).
