# Supplementary material for: Preservation of Metabolic Flexibility in Skeletal Muscle by a Combined Use of n-3 PUFA and Rosiglitazone in Dietary Obese Mice
Source: PLoS One. 2012 Aug 31;7(8):e43764. doi: 10.1371/journal.pone.0043764 (PMC3432031; doi:10.1371/journal.pone.0043764)
Supplement: Table S5 — Real-time quantitative RT-PCR analysis: genes and primers. (DOC) [file pone.0043764.s006.doc]

**Table S5** Real-time quantitative RT-PCR analysis: genes and primers

| **Gene symbol** | **Gene name** | **Accession number (RefSeq)** | **Forward primer (5’ – 3’)** | **Reverse primer (5’ – 3’)** |
| --- | --- | --- | --- | --- |
| *Acot1* | acyl-CoA thioesterase 1 | NM_012006.2 | AGCGCTGGCATGCACCTCCTG | TTCCCCAACCTCCAAACCATCATA |
| *CD36* | CD36 antigen | NM_001159558.1 | TCCTTGGCATGGTAGAGAT | ACCAAAGATGTAGCCAGTGTA |
| *Cpt1a* | carnitine palmitoyltransferase 1a, liver | NM_013495.2 | GCAGCTCGCACATTACAAGGACAT | ACTATGTGTCCTGTGGCGGGGGCT |
| *Cpt1b* | carnitine palmitoyltransferase 1b, muscle | NM_009948.2 | GGATGATGGCTACGGGGTCTCTTA | AGGGCAGCTGGGGTATCTCTTTTC |
| *Cyp1a1* | cytochrome P450, family 1, subfamily a | NM_009992.4 | TCCCCCACAGCACCACAAGAGATA | AGAGTGCCGCTGGGGGTGAGAAA |
| *eEf2* | eukaryotic translation elongation factor 2 | NM_007907.2 | GAAACGCGCAGATGTCCAAAAGTC | GCCGGGCTGCAAGTCTAAGG |
| *Fbp2* | fructose bisphosphatase 2 | NM_007994.3 | CCATGAGCCCGCTTCCCTTTGT | GGCGCTGTTCTGACCGTGACCT |
| *Glut4* | solute carrier family 2 (facilitated glucose transporter), member 4 | NM_009204.2 | ACCGGCTGGGCTGATGTGTCT | GCCGACTCGAAGATGCTGGTTGAATAG |
| *Gpd1* | glycerol-3-phosphate dehydrogenase 1 (soluble) | NM_005276.2 | GCAGACACCCAACTTTCGCATCA | CCGCCGCCTTGGTGTTGTCA |
| *Myh6* | myosin, heavy polypeptide 6, cardiac muscle, alpha | NM_010856.4 | TCCGTGCAGATAGAGATGAATAAG | CCCGCTGGAGGTTGTCG |
| *Myh7* | myosin, heavy polypeptide 7, cardiac muscle, beta | NM_080728.2 | GGCTAACCTGGAGAAGATGTGC | CCCGCTGGCTGGTGAGG |
| *Myl2* | myosin, light polypeptide 2, regulatory, cardiac, slow | NM_010861.3 | CCGAGGGCAAAGGGTCACTG | GGGGAAAGGCTGCGAACATCT |
| *Pdk4* | pyruvate dehydrogenase kinase, isoenzyme 4 | NM_013743.2 | GGCTTGCCAATTTCTCGTCTCTA | TTCGCCAGGTTCTTCGGTTCC |
| *Pgc1a* | peroxisome proliferative activated receptor, gamma, coactivator 1 | NM_008904.2 | CCCAAAGGATGCGCTCTCGTT | TGCGGTGTCTGTAGTGGCTTGATT |
| *Scd1* | stearoyl-Coenzyme A desaturase 1 | NM_009127.4 | ACTGGGGCTGCTAATCTCTGGGTGTA | GGCTTTATCTCTGGGGTGGGTTTGTTA |
| *Srebp1* | sterol regulatory element binding transcription factor 1 | NM_011480.3 | GCTTCCGGCCTGCTATGA | CAGGGCCTCGGTGTGCTC |
| *Tnnc1* | troponin C, cardiac/slow skeletal | NM_009393.2 | CGATGGCCGAATTGACTATGACGA | TGGGTGGAGGGGGAGAACAGG |
| *Trib3* | tribbles homolog 3 | NM_175093.2 | TTCCTACCGGGGCTGTCTTCTCTG | AGCCCGGCCTGATTTGTGGTC |
| *Ucp3* | uncoupling protein 3 (mitochondrial, proton carrier) | NM_009464.3 | AGAACCATCGCCAGGGAGGAAGGA | CACCGGGGAGGCCACCACTGT |
