# Supplementary material for: Preservation of Metabolic Flexibility in Skeletal Muscle by a Combined Use of n-3 PUFA and Rosiglitazone in Dietary Obese Mice
Source: PLoS One. 2012 Aug 31;7(8):e43764. doi: 10.1371/journal.pone.0043764 (PMC3432031; doi:10.1371/journal.pone.0043764)
Supplement: Table S6 — Real-time quantitative RT-PCR analysis in skeletal muscle. At the end of the experiment, mice were killed either without any additional manipulations, that is, while offered the ‘original’ cHF-based diets (OrD), or following the ‘diet-switch protocol’ when re-fed Chow diet; see Fig. 1. Control mice maintained on Chow diet throughout the intervention and killed in ad libitum fed state were also analyzed. Data are means±SE (n = 7–8). For gene symbols, see Table S5. a Significantly different from cHF, OrD. b significantly different from cHF+F, OrD. c significantly different from cHF+ROSI, OrD. d significantly different from cHF+F+ROSI, OrD. e Significantly different from cHF, Re-fed Chow. f significantly different from cHF+F, Re-fed Chow. g significantly different from cHF+ROSI, Re-fed Chow. h significantly different from cHF+F+ROSI, Re-fed Chow (Two-way ANOVA). i significantly different from Chow (t-test). (DOC) [file pone.0043764.s007.doc]

**Table S6** Real-time quantitative RT-PCR analysis in skeletal muscle

| **Gene symbol** | **Chow** | **cHF** | | **cHF+F** | | **cHF+ROSI** | | **cHF+F+ROSI** | |
| --- | --- | --- | --- | --- | --- | --- | --- | --- | --- |
|  |  | OrD | Re-fed Chow | OrD | Re-fed Chow | OrD | Re-fed Chow | OrD | Re-fed Chow |
| *Acot1* | 0.54 ± 0.06 | 1.00 ± 0.15^i^ | 1.00 ± 0.09^i^ | 1.28 ± 0.14^i^ | 0.66 ± 0.05^b^ | 1.12 ± 0.10^i^ | 0.73 ± 0.05^ci^ | 1.34 ± 0.11^i^ | 0.66 ± 0.08^d^ |
| *CD36* | 0.97 ± 0.08 | 1.00 ± 0.11^d^ | 1.19 ± 0.07 | 1.30 ± 0.10^i^ | 1.07 ± 0.06 | 1.19 ± 0.16 | 1.12 ± 0.07 | 1.51 ± 0.08^i^ | 1.17 ± 0.10^d^ |
| *Cpt1a* | 1.49 ± 0.31 | 1.00 ± 0.19 | 1.72 ± 0.37^a^ | 1.36 ± 0.22 | 1.37 ± 0.19 | 1.15 ± 0.28 | 1.15 ± 0.12 | 1.61 ± 0.15 | 1.86 ± 0.30 |
| *Cpt1b* | 0.69 ± 0.35 | 1.00 ± 0.13 | 2.18 ± 0.53^a^ | 0.69 ± 0.14 | 1.93 ± 0.30^bi^ | 1.05 ± 0.17 | 1.65 ± 0.29 | 1.30 ± 0.18 | 1.48 ± 0.41 |
| *Cyp1a1* | 0.92 ± 0.08 | 1.00 ± 0.03^d^ | 0.86 ± 0.05^h^ | 1.10 ± 0.07^d^ | 0.97 ± 0.05^h^ | 1.18 ± 0.08^d^ | 0.91 ± 0.041^h^ | 1.41 ± 0.08^i^ | 1.21 ± 0.06^di^ |
| *Fbp2* | 0.52 ± 0.11 | 1.00 ± 0.06^i^ | 0.89 ± 0.11 | 0.88 ± 0.13 | 0.85 ± 0.11 | 0.90 ± 0.10^i^ | 0.55 ± 0.10^c^ | 1.27 ± 0.06^i^ | 0.76 ± 0.11^d^ |
| *Glut4* | 1.68 ± 0.18 | 1.00 ± 0.13^i^ | 1.48 ± 0.14^a^ | 0.98 ± 0.08^i^ | 1.43 ± 0.08^b^ | 1.01 ± 0.09^i^ | 1.33 ± 0.11^c^ | 1.22 ± 0.12^i^ | 1.59 ± 0.11^d^ |
| *Gpd1* | 1.05 ± 0.09 | 1.00 ± 0.07 | 0.85 ± 0.06 | 0.84 ± 0.06^d^ | 0.97 ± 0.07 | 0.86 ± 0.10 | 0.90 ± 0.05 | 1.11 ± 0.05^b^ | 1.05 ± 0.07 |
| *Myh6* | 0.33 ± 0.09 | 1.00 ± 0.26 | 2.09 ± 0.33^i^ | 1.21 ± 0.54 | 1.48 ± 0.47^i^ | 1.25 ± 0.59 | 1.74 ± 0.55^i^ | 2.19 ± 0.46^i^ | 0.93 ± 0.31 |
| *Myh7* | 0.68 ± 0.11 | 1.00 ± 0.42 | 2.23 ± 0.26^ai^ | 1.05 ± 0.31 | 1.65 ± 0.36^i^ | 1.45 ± 0.60 | 1.47 ± 0.29^i^ | 1.81 ± 0.47^i^ | 1.30 ± 0.31 |
| *Myl2* | 1.17 ± 0.28 | 1.00 ± 0.23 | 1.93 ± 0.17^ai^ | 1.01 ± 0.19 | 2.44 ± 0.22^bi^ | 1.42 ± 0.31 | 2.46 ± 0.31^ci^ | 1.23 ± 0.20 | 1.43 ± 0.34^dfg^ |
| *Pdk4* | 0.41 ± 0.05 | 1.00 ± 0.13^i^ | 0.84 ± 0.13 | 1.29 ± 0.16^i^ | 0.53 ± 0.12^b^ | 1.03 ± 0.21 | 0.52 ± 0.06^c^ | 1.41 ± 0.16^i^ | 0.49 ± 0.04^d^ |
| *Pgc1a* | 0.81 ± 0.33 | 1.00 ± 0.19 | 0.91 ± 0.19 | 1.14 ± 0.33 | 1.30 ± 0.32 | 2.17 ± 0.72 | 0.82 ± 0.11^c^ | 1.40 ± 0.35 | 0.81 ± 0.20 |
| *Scd1* | 1.55 ± 0.30 | 1.00 ± 0.09 | 1.15 ± 0.14 | 0.59 ± 0.08^ci^ | 0.64 ± 0.08^i^ | 1.52 ± 0.32^d^ | 1.02 ± 0.07^c^ | 0.75 ± 0.11^i^ | 0.73 ± 0.15^i^ |
| *Srebp1* | 2.79 ± 0.43 | 1.00 ± 0.12^i^ | 1.70 ± 0.35^ai^ | 0.85 ± 0.11^i^ | 1.35 ± 0.21^i^ | 1.10 ± 0.16 | 2.09 ± 0.29^c^ | 1.24 ± 0.18 | 1.38 ± 0.18^i^ |
| *Tnnc1* | 0.23 ± 0.07 | 1.00 ± 0.28 | 1.15 ± 0.16^i^ | 1.12 ± 0.33 | 0.97 ± 0.19^i^ | 1.27 ± 0.32 | 1.42 ± 0.42 | 1.73 ± 0.34^i^ | 0.74 ± 0.15^d^ |
| *Trib3* | 1.51 ± 0.14 | 1.00 ± 0.08^i^ | 1.06 ± 0.11^i^ | 1.09 ± 0.07^i^ | 1.81 ± 0.24^be^ | 0.98 ± 0.05^i^ | 1.41 ± 0.06^c^ | 1.56 ± 0.09^ac^ | 2.09 ± 0.15^degi^ |
| *Ucp3* | 0.88 ± 0.23 | 1.00 ± 0.12 | 1.21 ± 0.31 | 1.02 ± 0.10 | 1.00 ± 0.17 | 0.98 ± 0.21 | 0.81 ± 0.12 | 1.14 ± 0.10 | 1.45 ± 0.17 |

At the end of the experiment, mice were killed either without any additional manipulations, that is, while offered the 'original' cHF-based diets (OrD), or following the 'diet-switch protocol' when re-fed Chow diet; see Fig. 1. Control mice maintained on Chow diet throughout the intervention and killed in ad libitum fed state were also analyzed. Data are means±SE (*n*=7–8). For gene symbols, see Table S5.

^a^ Significantly different from cHF, OrD.

^b^ significantly different from cHF+F, OrD.

^c^ significantly different from cHF+ROSI, OrD.

^d^ significantly different from cHF+F+ROSI, OrD.

^e^ Significantly different from cHF, Re-fed Chow.

^f^ significantly different from cHF+F, Re-fed Chow.

^g^ significantly different from cHF+ROSI, Re-fed Chow.

^h^ significantly different from cHF+F+ROSI, Re-fed Chow (Two-way ANOVA).

^i^ significantly different from Chow (t-test).
