# Supplementary material for: Preservation of Metabolic Flexibility in Skeletal Muscle by a Combined Use of n-3 PUFA and Rosiglitazone in Dietary Obese Mice
Source: PLoS One. 2012 Aug 31;7(8):e43764. doi: 10.1371/journal.pone.0043764 (PMC3432031; doi:10.1371/journal.pone.0043764)
Supplement: Supporting Information S1 — (DOC) [file pone.0043764.s008.doc]

**Supporting Information**

*Expression profiling using microarrays*

The Two-color whole mouse genome (4 x 44 k) Agilent oligo microarray kits (G4122F, Agilent Technologies, Inc. Santa Clara, CA) were used to analyse gene expression in individual mice. Preparation of samples and the microarray hybridization were performed according to the manufacturer’s protocol with a few modifications as described previously [1] and with arrays rotating at 10 rpm. Briefly, cDNA was synthesized for each animal using 1 μg of gastrocnemius muscle total RNA using the Agilent Low-RNA Input Fluorescent Linear Amplification kit without addition of spikes. In the first study, individual samples from the cHF, cHF+F, and cHF+ROSI groups were used; in the latter case, we also included samples from another group of mice, subjected to an intervention using a higher dose of rosiglitazone admixed to the cHF diet (i.e., 100 mg rosiglitazone/kg diet) for the reference pool, but these samples were excluded for data analysis in this study. In the second study, only cHF+ROSI and cHF+F+ROSI were analyzed. In both studies, samples were split in two equal amounts, to synthesize cyanine 3-cytidine triphosphate (Cy3) and cyanine 5-cytidine triphosphate (Cy5) labeled cRNA using half the amounts per dye as indicated by the manufacturer. Labeled cRNA was purified using RNeasy columns (Qiagen, Venlo, The Netherlands). Cy3 and Cy5 concentration, A_260_/A_280_ ratio and cRNA concentration were determined using the NanoDrop spectrophotometer (IsoGen Life Science, Maarsen, The Netherlands) and yield and specific activity of each reaction were calculated according to the manufacturer’s protocol. Overall, thirty-nine of the forty samples met the criteria of cRNA yield higher than 825 ng and the specific activity higher than 8 pmol Cy3 or Cy5 per μg cRNA (the sample that did not met the criteria was excluded from further analysis). Then, all Cy3-labeled cRNA samples were pooled on an equimolar basis and subsequently used as a common reference pool. Individual 825 ng Cy5-labeled cRNA and 825 ng pooled Cy3-labeled cRNA were fragmented in 1x fragmentation and 1x blocking agent at 60°C for 30 min, afterward mixed with GEx Hybridization Buffer HI-RPM and hybridized in the Agilent Microarray Hybridization Chamber rotating at 10 rpm at 65°C for 17 hours. After hybridization, slides were washed according to the wash protocol. Arrays were scanned with an Agilent scanner with 10% and 100% laser-power intensities. All used chemicals and instruments were by Agilent Technologies, unless otherwise stated.

*Microarray data analysis*

Per study, signal intensities for each spot were quantified using Feature Extraction software version 10.5.1.1 (Agilent Technologies, Inc. Santa Clara, CA). Quality control of each microarray was performed visually using quality control graphs from Feature Extraction and M-A plots and boxplots using limmaGUI in R (Bioconductor, Seattle, WA, USA). After quality control, data from all arrays were imported into GeneMaths XT 2.12 (Applied Maths, Sint-Martens-Latem, Belgium). Median spot values with a mean Cy-5 and Cy-3 signal/background ratio over all arrays lower than two were excluded; remaining data were log-transformed. The log-transformed Cy5 signal was normalized against Cy3 intensities as described before [2]. Since cHF+ROSI was used for both microarray studies, we analysed coherence between the hybridisation studies. This indicated that data are very reproducible (data not shown), as reported previously [3]. Pathway analysis was performed using MetaCore (GeneGo, Carlsbad, CA, USA), GO overrepresentation analysis, and mining of scientific literature.

**References**

1. van Helden YG, Godschalk RW, Heil SG, Bunschoten A, Hessel S, Amengual J, Bonet ML, von LJ, van Schooten FJ, Keijer J (2010) Downregulation of Fzd6 and Cthrc1 and upregulation of olfactory receptors and protocadherins by dietary beta-carotene in lungs of Bcmo1-/- mice. Carcinogenesis 31: 1329-1337.

2. Pellis L, Franssen-van Hal NL, Burema J, Keijer J (2003) The intraclass correlation coefficient applied for evaluation of data correction, labeling methods, and rectal biopsy sampling in DNA microarray experiments. Physiol Genomics 16: 99-106.

3. van Schothorst EM, Pagmantidis V, de Boer VC, Hesketh J, Keijer J (2007) Assessment of reducing RNA input for Agilent oligo microarrays. Anal Biochem 363: 315-317.
